# Genome-Wide Methylation Patterns in *Salmonella enterica* Subsp. *enterica* Serovars

**DOI:** 10.1371/journal.pone.0123639

**Published:** 2015-04-10

**Authors:** Cary Pirone-Davies, Maria Hoffmann, Richard J. Roberts, Tim Muruvanda, Ruth E. Timme, Errol Strain, Yan Luo, Justin Payne, Khai Luong, Yi Song, Yu-Chih Tsai, Matthew Boitano, Tyson A. Clark, Jonas Korlach, Peter S. Evans, Marc W. Allard

**Affiliations:** 1 Division of Microbiology, Office of Regulatory Science, Center for Food Safety and Applied Nutrition, U.S. Food and Drug Administration, College Park, Maryland, United States of America; 2 New England Biolabs, Ipswich, Massachusetts, United States of America; 3 Office of Analytics and Outreach, Center for Food Safety and Applied Nutrition, U.S. Food and Drug Administration, College Park, Maryland, United States of America; 4 Pacific Biosciences, Menlo Park, California, United States of America; Universität Stuttgart, GERMANY

## Abstract

The methylation of DNA bases plays an important role in numerous biological processes including development, gene expression, and DNA replication. *Salmonella* is an important foodborne pathogen, and methylation in *Salmonella* is implicated in virulence. Using single molecule real-time (SMRT) DNA-sequencing, we sequenced and assembled the complete genomes of eleven *Salmonella enterica* isolates from nine different serovars, and analysed the whole-genome methylation patterns of each genome. We describe 16 distinct N^6^-methyladenine (m6A) methylated motifs, one N^4^-methylcytosine (m4C) motif, and one combined m6A-m4C motif. Eight of these motifs are novel, i.e., they have not been previously described. We also identified the methyltransferases (MTases) associated with 13 of the motifs. Some motifs are conserved across all *Salmonella* serovars tested, while others were found only in a subset of serovars. Eight of the nine serovars contained a unique methylated motif that was not found in any other serovar (most of these motifs were part of Type I restriction modification systems), indicating the high diversity of methylation patterns present in *Salmonella*.

## Introduction

The methylation of DNA is important in all kingdoms of life as a mechanism of epigenetic control [1–3]. Methylation is achieved through the action of methyltransferase enzymes (MTases), which covalently attach methyl groups to DNA bases. In eukaryotes, 5-methylcytosine (m5C) is the most common methylation. In contrast, N^6^-methyladenine (m6A) is the most frequent methylation in prokaryotes, although N^4^-methylcytosine (m4C) and m5C are also widespread.

Methylation in eukaryotes has been well studied and is known to mediate diverse processes including growth, development, and disease [4]. In prokaryotes, methylation is a key component of restriction-modification (RM) systems, which protect cells from foreign DNA. RM systems are composed of multiple proteins, including at least one MTase, which recognizes and methylates a base contained within a specific sequence motif, and one endonuclease, or REase, which cleaves foreign DNA with a methylation pattern different from that of the host DNA. RM systems are subdivided into four main classes that differ in subunit composition, motif characteristics, cofactor requirements, and location of DNA cleavage (for review, see [5]). In brief, Type 1 RM systems are composed of two restriction subunits (R), two methylation subunits (M) and one specificity subunit (S), which recognizes specific DNA sequences. Recognized motifs are asymmetric and bipartite. Type II systems include one R and one M subunit which can function independently, and recognized motifs are mostly symmetric. Type III systems are hetero-oligomers composed of a mod subunit (recognizes and modifies DNA) and a res subunit that is only active in a mod-res complex. The only RM systems that recognize methylated, instead of unmethylated sites, are Type IV. Methylation in bacteria also influences critical processes including gene regulation, cell cycle control, pathogenicity, and DNA repair [2].

Despite the important implications of bacterial methylation, its distribution, diversity, and functional consequences have not been extensively investigated. This paucity of data can, in part, be attributed to technological limitations. Methylation studies in eukaryotes have been facilitated by the development of detection methods for m5C, including bisulfite conversion, which allows for genome-wide modification analyses. Comparable methods have not been available for the detection of m6A and m4C until recent advances in sequencing technology. SMRT sequencing couples whole-genome sequencing with the simultaneous detection of base modifications using kinetic signals during DNA polymerization [6, 7]. This new technology has led to insights regarding the methylomes of several bacterial species [8–12]. However, methylation is widespread throughout the bacterial kingdom and is very diverse [13]. Thus, more studies are needed to gain a comprehensive understanding of the distribution and diversity of methylation motifs and their associated MTases, and ultimately to comprehend methylation functions and evolutionary history in these organisms.

*Salmonella enterica* is the leading cause of death and hospitalizations due to foodborne pathogens each year [14]. Previous studies have shown that the methylation of the G^m6^ATC motif by the MTase Dam is an essential factor in the virulence of *Salmonella*, and that a lack of methylation leads to attenuation in animal models [15]. Subsequent studies have elucidated the mechanisms by which some virulence genes are regulated by Dam, including the plasmid-encoded fimbriae (pef) locus [16] and the std fimbrial operon [17]. In addition, Dam regulates both the phase variation of STM2209-STM2208 which alters lipopolysaccharide O-antigen side chain length [18], and the phase variation of the phage P22 glucosyltransferase (gtr) operon which controls O-antigen glucosylation [19]. Thus, it is possible that the methylation of other motifs in *Salmonella* also may have implications for virulence, pathogenicity, and other functions. Here, we sequenced and closed the genomes of six *Salmonella enterica* isolates from five serovars. We then analysed their methylomes, along with the methylomes of four additional serovars that we sequenced previously [11, 20–22], and employed a bioinformatics approach to identify methyltransferases and match them to observed methylated motifs in the genomes. We also examined how methylation patterns varied between *Salmonella* serovars.

## Materials and Methods

We selected five serovars of *Salmonella enterica* subs *enterica* from our in-house strain collection at the FDA-CFSAN. These included *Salmonella enterica* subs *enterica* serovar (*S*. Bareilly), *S*. Abaetetuba, *S*. Abony, *S*. Anatum, *S*. Bredeney, *S*. Montevideo, and two isolates of *S*. Enteritidis. We also included data from four serovars we sequenced previously, *S*. Javiana, *S*. Typhimurium, *S*. Heidelberg, and *S*. Cubana [11, 20–22] (see Table 1 for strain names and accession numbers).

**Table 1:** Summary of *Salmonella* genomes sequenced in this study.

Serovar	Chromosome size (bp)	Plasmid size (bp)	GenBank Accession (chromosome)	GenBank Accession (plasmid)	Phage	MTase on phage (specificity, if known)	MTase on plasmid (specificity, if known)
*S*. Bareilly CFSAN000189	4730612	78193	CP006053.1	CP006054.1	Salmon_Fels_1_NC_010391 Gifsy_1_NC_010392	_	M.SbaUORF280P
*S*. Abony CFSAN001275	4737447	NA	CP007534.1	_	Entero_ST64T_NC_004348 Gifsy_2_NC_010393	_	_
*S*. Anatum CFSAN000665	4706101	NA	CP007531.1	_	Salmon_Fels_1_NC_010391 Gifsy_1_NC_010392	M.SenAnaORF14155P	_
*S*. Cubana CFSAN002050	4977480	166,668 122,863	CP006055.1	CP006056.1 CP006057.1	Gifsy_1_NC_010392 Salmon_vB_SemP_Emek_NC_018275	_	**M.Sen2050ORF235P**(GATC) M.Sen2050ORF245P M.Sen2050ORF400P M.Sen2050ORF480P(CAGCTG)
*S*. Heidelberg CFSAN002069	4783943	110,363 37,679	CP005390.2	CP005389.2 CP005391.2	Entero_P22_NC_002371 Gifsy_2_NC_010393	**M.Sen2069ORF4005P** (GATC)	M.Sen2069ORF23325P
*S*. Heidelberg CFSAN002064	4783867	37692	CP005995.1	CP005994.1	Entero_P22_NC_002371 Gifsy_2_NC_010393	**M.Sen2069ORF21380P** (GATC)	_
*S*. Javiana CSFAN001992	4634161	24,012 17,094	CP004027.1	CP004026.1 CP004028.1	Gifsy_2_NC_010393 Salmon_RE_2010_NC_019488 Entero_PsP3_NC_005340	M.SenJORF19790P (GATC)	_
*S*. Montevideo CFSAN000255	4694375	NA	CP007530.1	_	Salmon_vB_SosS_Oslo_NC_018279 Entero_Fels_2_NC_010463	**M.Sen255II** (ATGCAT)	_
*S*. Enteritidis CFSAN000158	4679662	59369	CP007528.1	CP007529.1	Salmon_RE_2010_NC_019488 Gifsy_2_NC_010393	**M.Sen158III** (GATC)	_
S. Enteritidis CFSAN000111	4679081	39599	CP007598.1	CP007599.1	Gifsy_2_NC_010393 Salmon_RE_2010_NC_019488	M.Sen1427ORF7910P (GATC)	_
S. Typhimurium CFSAN001921	4859931	3,609 4,675 221,009	CP006048.1	CP006052.1 CP006051.1 CP006050.1	Salmon_ST64B_NC_004313 Gifsy_1_NC_010392 Gifsy_2_NC_010393 Entero_ST104_NC_005841	_	M.SenTFORF23885P (CAGCTG) M.SenTFORF24805P (CCNGG)

Each strain was plated onto Trypticase Soy Agar and incubated overnight at 37°C. Cells were then inoculated into Trypticase Soy Broth for DNA extraction. A 1 ml-aliquot was pelleted, and genomic DNA was extracted using the DNeasy Blood and Tissue kit from Qiagen (Qiagen, CA, USA). All samples were analyzed at the exponential stage of growth.

DNA was sheared to approximately 10 kb using a Covaris g-TUBE (Covaris, Inc.; Woburn, MA). SMRTbell 10 kb template libraries were prepared using DNA Template Prep Kit 2.0 and the Low-Input 10 kb Library Protocol (Pacific Biosciences; Menlo Park, CA, USA). In brief, DNA was concentrated, repaired, ligated to hairpin adapters, and purified. Incompletely formed SMRTbell templates were digested with a combination of Exonucleases III and VII. Adapters were annealed, and SMRT sequencing was carried out on the PacBioRS II (Pacific Biosciences; Menlo Park, CA, USA) using standard protocols.

Analysis of sequence reads was implemented using SMRT Analysis 1.10 and the SMRT Portal 2.0 platform (Pacific Biosciences). *De novo* assembly was performed using the Hierarchical Genome Assembly Process (HGAP) with default parameters [23]. HGAP consists of three steps to ensure high accuracy. First, Basic Local Alignment with Successive Refinement (BLASR) is used to align all reads to the longest seed reads and a consensus is generated to create pre-assembled reads. Preassembled reads are then assembled using the Celera assembler. Finally, all reads are mapped to the *de novo* assembly and final consensus and accuracy scores are determined using the Quiver consensus algorithm. HGAP outputs assemblies with overlapping regions at the ends. Coordinates of this region were identified using dot plots in Gepard [24], and trimmed from one end to circularize the genome. Genomes were checked manually for even sequencing coverage. Genomes were annotated using the NCBI (National Center for Biotechnology Information) Prokaryotic Genomes Automatic Annotation Pipeline [25] (http://www.ncbi.nlm.nih.gov/genomes/static/Pipeline.html). Prophages were detected using PHAST [26]. Only prophages scored as intact are reported here. We excluded putative intact prophages that did not show significant sequence similarity to known phages using the Basic Local Alignment Search Tool (BLAST) sequence alignment tool with default parameters.

Motif Detection and Analysis was also carried out using SMRT Analysis 1.1 and the RS_Modification_and_Motif_Analysis.1 protocol as described at http://www.pacb.com/pdf/TN_Detecting_DNA_Base_Modifications.pdf. Interpulse durations (IPDs) were measured based on the kinetic signals [7] and processed as described previously [6]. At each position in the genome, the observed IPD was compared to the IPD of an in-silico control using a two-sample t-test, and a QV score was calculated as QV = -10 log (p-value). Bases were accepted as modified based on a minimum QV threshold value. QV 30 was used as a threshold for preliminary analyses. A plot of QV versus coverage was then constructed using publicly available R scripts found at: https://github.com/PacificBiosciences/motif-finding. The observed bimodal distribution of kinetic data, resulting from modified and unmodified positions, was then used to determine a more stringent QV threshold (S1 Fig). Only sites with a minimum of 25x coverage were included. Motifs were identified using the algorithm MotifMaker. m6A and m4C motifs can be reliably detected with 25x coverage across all positions in the genome, but m5C requires either significantly higher coverage (~100x) or Tet-methylation for confident detection. In this study we report only m6A and m4C methylations. To identify MTases, assembled genomes were scanned for homologs of RM system genes using in-house software (e value > 1e-11) to identify putative MTases as previously described [10]. Predicted specificities were assigned to candidate MTases based on specificities of the known MTases. The presence of functional motifs and information regarding the placement of the gene within the genome were also used to support or reject those assignments, as were known characteristics of different MTase types. For example, Type III MTases and most Type IIG systems only methylate one strand of their recognition sequence, whereas Type I systems have bipartite recognition sequences. MTase candidates with predicted specificities were matched where possible with observed motifs found in our motif analyses. If a single candidate MTase existed for an observed motif, then that gene was assumed to be responsible for that particular specificity. If multiple candidates existed for a single motif, no MTase was assigned. When making assignments of new motifs to specific MTases, we always cross-checked the matched gene against other similar genes in REBASE and against the unassigned motifs from the more than 700 other genomes for which we have PacBio data. In many cases, the same motif occurred in a different genome with an essentially identical methyltransferase or specificity subunit protein sequence, adding weight to the strength of the assignment. Raw processed PacBio data files were deposited in the Sequence Read Archive (SRA) database of the National Center for Biotechnology Information (NCBI) (http://www.ncbi.nlm.nih.gov/sra) (S2 Table) and MTase information and sequences were deposited in REBASE (http://rebase.neb.com/rebase/rebase.html).

## Results and Discussion

### Genome Assemblies

All genomes were assembled into a single, circular chromosomal contig and up to three plasmids. Consensus accuracy scores were at least 99.9999% for all assemblies. Sizes of *Salmonella* chromosomes ranged from 4,547,600 – 4,977,480 bp, plasmid sizes ranged from 3,609–221,009 bp (Table 1). Sequences were deposited in GenBank. Putative prophages and BLAST alignment data are reported in Table 1.

### Methylation Patterns

**Fig 1:**
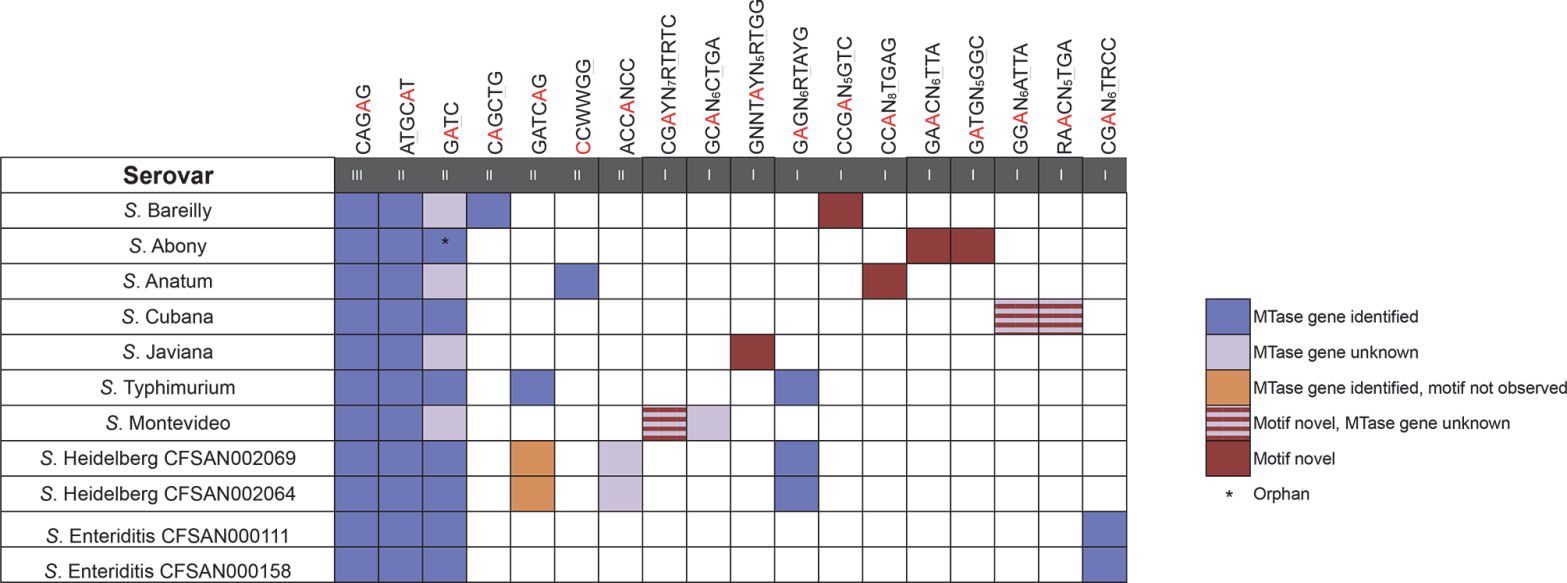
The methylomes of eleven *Salmonella* serovars. Violet = MTase identified, light violet = MTase unknown, red = novel motif, orange = MTase present in genome, motif not observed. Light violet/red stripe = novel motif, MTase unknown. Roman numerals indicate MTase type (I – III). Note the majority of novel MTases are Type I systems.

This is the first comparative report of genome-wide methylation patterns in the pathogenic bacteria *Salmonella enterica*. We analyzed the methylomes of five *Salmonella enterica* subsp. *enterica* serovars, including two isolates of *S*. Enteritidis. We also sequenced and released their closed genomes. We present those results, along with data from four additional *Salmonella* serovars, *S*. Javiana, *S*. Typhimurium, *S*. Heidelberg, and *S*. Cubana, which we analyzed previously [11, 20–22]. In total, we observed 18 motifs among the nine *Salmonella* serovars, 16 m6A motifs, one m4C motif, ^m4^CCWWGG, and one Type I MTase which encodes both m6A and m4C activities, G^m6^ATGN_5_G^4m^ GC (Fig 1; an underscore represents the base which is methylated in the opposite DNA strand; W = A or T). Eight of the motifs were novel, i.e., they have not been previously observed in any bacterial species. We were able to match 13 of the *Salmonella* motifs to their respective MTase enzymes in most of the serovars tested (S1 Table).

Several motifs were common among multiple serovars, while other motifs were unique to specific serovars. All *Salmonella* serovars examined contained the methylated motifs ATGC^m6^AT, CAG^m6^AG, and G^m6^ATC. In all serovars, we identified a Type III MTase responsible for the methylation of CAG^m6^AG, and an extremely common Type II MTase was found to methylate the ATGC^m6^AT motif (see Table 2 for a list of enzyme names specific to each strain). The methylation of ATGC^m6^AT was never complete (38–78.5%). This MTase is usually active in *Salmonella*, although rarely active in *E*. *coli*, and is not thought to be an essential gene [27]. Confident assignment of an MTase to the G^m6^ATC motifs could only be performed in eight of the eleven isolates: two were orphan MTases, and the remaining were common Type II enzymes. In multiple serovars, we identified candidate enzymes that have the potential to methylate this motif (Table 3).

**Table 2:** Summary of motifs and methyltransferases found in each *Salmonella* genome.

Serovar	Enzyme Assignment	Gene Locus_Tag (GenBank)	Type	Sub- Type	Motif Observed	Motif Uni-que*	% Methyl-ated 5'-3'/3'-5'	Number Methyl- ated Motifs (5'-3' strand/ 3'-5' strand)	Number Motifs in Genome (5'-3' strand/ 3'-5' strand)
*S*. Bareilly CFSAN000189	M.SbaUI	SEEB0189_17520	III	beta	CAG^m6^AG	no	97.7	5652	5787
	M.SbaUII	SEEB0189_19945	II	beta	C^m6^AGCTG	no	88.2	1466	1662
	M.SbaUIII	SEEB0189_19740	I	gamma	CCG^m6^ANNNNNGTC	yes	98.6/ 98.6	482/ 482	489/ 489
	M.SbaUIV	SEEB0189_02925	II	beta	ATGC^m6^AT	no	78.5	1093	1392
	M.SbaUDam	SEEB0189_02450	Orphan	_	G^6m^ATC	no	98.6/ 98.6	37148	37688
*S*. Abony CFSAN001275	M.SenAboI	SEEA0014_11325	III	beta	CAG^m6^AG	no	96.8	5391	5569
	M.SenAboII	SEEA0014_03225	II	beta	ATGC^m6^AT	no	38	283	744
	M.SenAboIV	SEEA0014_08865	I	gamma	GA^m6^ACNNNNNNNTTA	yes	94.9/ 93.5	410/ 404	432/ 432
	M.SenAboDam	SEEA0014_03700	Orphan	alpha	G^6m^ATC	no	95.1/ 95.1	35607	37436
	M1.SenAboIII	SEEA0014_08700	I	gamma	G6mATGNNNNNG4mGC/ G4mCCNNNNNCATC	yes	96.1/ 31.0	1260/ 406	1311/ 1311
	M2.SenAboIII	SEEA0014_08705	I	gamma	G6mATGNNNNNG4mGC/ G4mCCNNNNNCATC	yes	96.1/ 31.0	1260/ 406	1311/ 1311
*S*. Anatum CFSAN000665	M.SenAnaI	SEEA1592_11695	I	gamma	CC^m6^ANNNNNNNNTGAG	yes	99.7/ 99.4	354/ 353	355/ 355
	M.SenAnaII	SEEA1592_09525	III	beta	CAG^m6^AG	no	100.0	5509	5511
	M.SenAnaIII	SEEA1592_17520	II	beta	ATGC^m6^AT	no	66.7/ 66.7	674	1010
	M.SenAnaIV	SEEA1592_11855	II	beta	^4m^CCWWGG	no	83.3/ 83.3	1423	1708
	M.SenAnaDam	SEEA1592_01330	Orphan	_	G^6m^ATC	no	99.8/ 99.8	37140	37224
*S*. Cubana CFSAN002050	M.Sen2050I	CFSAN002050_08375	III	beta	CAG^m6^AG	no	95.1	6235	6558
	M.Sen2050II	CFSAN002050_23900	II	beta	ATGC^m6^AT	no	45.1/ 45.1	510	1131
	_	_	I	_	GG^m6^ANNNNNNATTA	yes	92.7/ 92.3	459/ 457	495/ 495
	_	_	I	_	TC^m6^ANNNNNGTTY	yes	95.5/ 92.3	1248/ 1338	1352/ 1352
*S*. Heidelberg CFSAN002064	M.Sen2064I	CFSAN002064_15765	I	gamma	G^m6^AGNNNNNNRTAYG	no	97.9/ 97.5	231/ 230	236/ 236
	M.Sen2064II	CFSAN002064_18310	III	beta	CAG^m6^AG	no	98.2	5587	5691
	M.Sen2064III	CFSAN002064_10125	II	beta	ATGC^m6^AT	no	42.4	319	752
	_	_	II	_	ACC^m6^ANCC	no	99.4	2703	2719
*S*. Heidelberg CFSAN002069	M.Sen2069I	CFSAN002069_07060	III	beta	CAG^m6^AG	no	97.9	5816	5939
	M.Sen2069II	CFSAN002069_09575	I	gamma	G^m6^AGNNNNNNRTAYG	no	97.5/ 97.9	238/ 239	244/ 244
	M.Sen2069III	CFSAN002069_15235	II	beta	ATGC^m6^AT	no	42.2/ 42.2	217	514
	_	_			ACC^m6^ANCC		99	2747	2774
*S*. Javiana CFSAN001992	M.SenJI	CFSAN001992_09405	III	beta	CAG^m6^AG	no	97.8	5410	5523
	M.SenJII	CFSAN001992_11490	I	gamma	CC^m6^AYNNNNNRTANNC	yes	98.1/ 97.7	474/ 472	483/ 483
	M.SenJIII	CFSAN001992_16620	II	beta	ATGC^m6^AT	no	58.8/ 58.8	803	1364
	_	_			G^6m^ATC	no	98.9/ 98.9	36330	36738
*S*. Montevideo CFSAN000255	M.Sen255I	Y007_00590	III	beta	CAG^m6^AG	no	99.4	5504	5535
	M.Sen255II	Y007_12075	II	beta	ATGC^m6^AT	no	50.0/ 50.0	387	774
	_	_	I	_	CG^6m^AYNNNNNNNRTRTC	yes	99.1/ 98.9	439/ 438	443/ 443
	_	_	II	alpha	G^6m^ATC	no	99.1/ 99.1	36866	37204
	_	_	I	_	GC^m6^ANNNNNNCTGA	no	98.6/ 99.5	554/ 559	562/ 562
*S*. Enteriditis CSFAN000111	M.Sen1427II	SEEE1427_7355	I	gamma	CG^m6^ANNNNNNTRCC	no	98.4/ 97.9	1721/ 1712	1749/ 1749
	_	_	II		G^6m^ATC	no	98.8/ 98.8	36824	37256
	M.Sen1427I	SEEE1427_9465			CAG^m6^AG	no	99.2	5505	5549
	M.Sen1427III				ATGC^6m^AT	no	43	315	732
S. Enteriditis CFSAN000158	M.Sen158I	SEEE0968_18850	III	beta	CAG^m6^AG	no	98.1	5490	5599
	M.Sen158II	SEEE0968_20955	I	gamma	CG^m6^ANNNNNNTRCC	no	99.0/ 98.0	1739/ 1722	1757/ 1757
	M.Sen158III	SEEE0968_03950	II	beta	ATGC^6m^AT	no	41.3/ 41.3	302	732
S. Typhimurium CFSAN001921	M.SenTFI	CFSAN001921_15255	III	beta	CAG^m6^AG	no	89.3	5635	6308
	M.SenTFII	CFSAN001921_17800	I	gamma	CRT^m6^AYNNNNNNCTC	no	90.7/ 89.1	233/ 229	257/ 257
	M.SenTFIII	CFSAN001921_00055	II	beta	ATGC^m6^AT	no	60.9	630	1035
	SenTFIV	CFSAN001921_17955	II	alpha	GATC^6m^AG	no	94.3	2841	3011

*A unique motif refers to one that has not been previously observed in any bacterial species.

**Table 3:** Methyltransferases identified in the *Salmonella* serovars, but not assigned to a motif.

Serovar	Enzyme Assignment	Type	SubType	Motif (if known)
*S*. Bareilly CFSAN000189	M.SbaUORF19730P	I	gamma	-
	M.SbaUORF280P	II	beta	-
*S*. Abony CFSAN001275	M.SenAboORF8720P	I	gamma	-
*S*. Anatum CFSAN000665	M.SenAnaDamP	Orphan	alpha	G^6m^ATC
	M.SenAnaORF14155P	II	alpha	G^6m^ATC
*S*. Cubana CFSAN002050	M.Sen2050DamP	Orphan	alpha	G^6m^ATC
	M.Sen2050ORF235P	II	alpha	G^6m^ATC
	M.Sen2050ORF245P	II	gamma	-
	M.Sen2050ORF400P	II	gamma	-
	M.Sen2050ORF480P	II	beta	-
	M.Sen2050ORF4940P	I	gamma	-
	M.Sen2050ORF5885P	I	gamma	-
*S*. Heidelberg CFSAN002064	M.Sen2064DamP	Orphan	alpha	G^6m^ATC
	M.Sen2064ORF21380P	II	alpha	G^6m^ATC
	Sen2064ORF15615P	II	G,S	GATC^6m^AG
*S*. Heidelberg CFSAN002069	M.Sen2069DamP	Orphan	alpha	G^6m^ATC
	M.Sen2069ORF4005P	II	alpha	G^6m^ATC
	M.Sen2069ORF23325P	II	beta	-
	Sen2069ORF9735P	II	G,S	GATC^6m^AG
*S*. Javiana CFSAN001992	M.SenJORF11520P	I	gamma	-
	M.SenJDamP	orphan	alpha	G^6m^ATC
	M.SenJORF19790P	II	alpha	G^6m^ATC
	M.SenJORF20475P	II	alpha	G^6m^ATC
	M.SenJORF6415P	II		G^6m^ATC
*S*. Montevideo CFSAN000255	M.Sen255DamP	Orphan	alpha	G^6m^ATC
	M.Sen255ORF17075P	II	alpha	G^6m^ATC
	M.Sen255ORF20925P	I	gamma	-
	M.Sen255ORF5995P	I	gamma	-
*S*. Enteritidis CSFAN000111	M.Sen1427DamP	Orphan	alpha	G^6m^ATC
	M.Sen1427ORF7380P	I	gamma	-
	M.Sen1427ORF7910P	II	alpha	G^6m^ATC
S. Enteritidis CFSAN000158	M.Sen158DamP	Orphan	alpha	G^6m^ATC
	M.Sen158ORFDP	II	alpha	G^6m^ATC
	M.Sen158ORF20930P	I	gamma	-
S. Typhimurium CFSAN001921	M.SenTFDamP	Orphan	alpha	G^6m^ATC
	M.SenTFORF6885P	II		G^6m^ATC
	M.SenTFORF23885P	II	beta	C^m6^AGCTG
	M.SenTFORF24320P	II		-
	M.SenTFORF3520P	III	beta	-

^a^m5C MTases not included.

**Fig 2:**
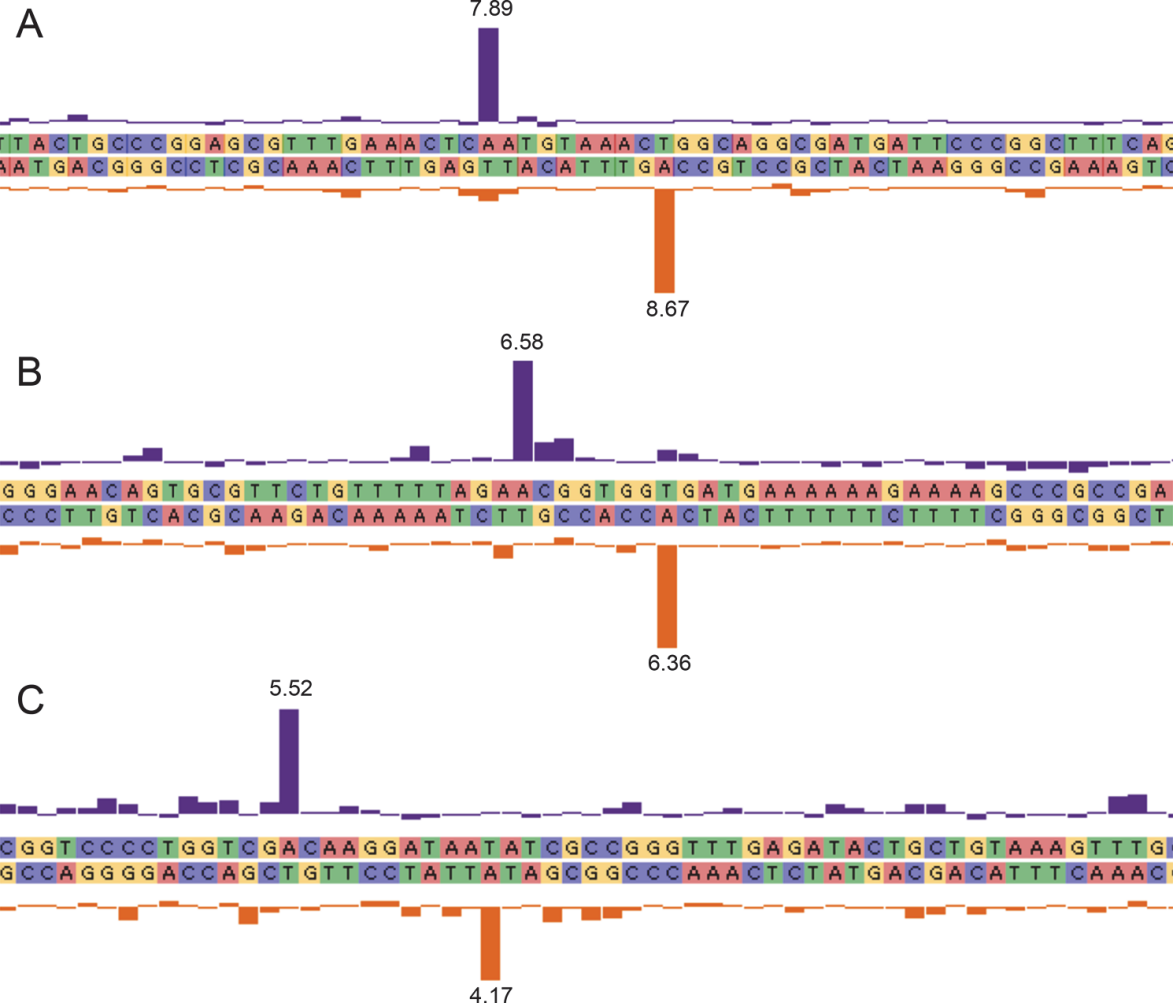
Diagram of the interpulse duration (IPD) ratio of three novel motifs identified in three *Salmonella* serovars. Vertical axis = IPD ratio, horizontal axis = genome position. IPD ratio listed next to bar. A. Motif CC^m6^AN_8_ TGAG in *S*. Anatum. B. Motif RA^m6^ACN_5_ TGA in *S*. Cubana. C. Motif CG^m6^AYN_6_RTRTC in *S*. Montevideo.

Other observed motifs were common among a subsection of the serovars examined. For example *S*. Typhimurium and both isolates of *S*. Heidelberg contained the common motif G^m6^AGN_6_RTAYG that is methylated by a Type I MTase. Six of the nine serovars, *S*. Bareilly, *S*. Abony, *S*. Cubana, *S*. Javiana, *S*. Montevideo, and *S*. Anatum, contained a motif not found in the other serovars tested (Fig 1). For example, in *S*. Anatum, we observed the motif CC^m6^AN_7_ TGAG. Fig 2 shows the kinetic signals of three of these motifs. In most cases these unique motifs were strongly methylated. Several novel motifs were not matched to any MTases including GG_m6_AN_6_ATTA and RA_m6_ACN_5_ TGA in *S*. Cubana, and CG_m6_AYN_7_RTRTC in *S*. Montevideo.

Several observed motifs could not be assigned to a single MTase. In some cases, there were multiple MTases with predicted specificities that matched that of an observed motif. In these cases, it was not possible to predict which enzyme was responsible for the methylation of the observed motifs, and thus no enzyme was assigned. Furthermore, we could not rule out the possibility that multiple enzymes methylated the same motif, as has been observed with G^m6^ATC [28]. MTases may also be promiscuous [29], i.e., they methylate multiple motifs, making a match to any single motif unrealistic. In some cases, there was no MTase present in the genome with a specificity predicted to recognize an observed methylated motif.

On other occasions, we did not observe the methylation of a motif that we predicted would be present based on a putative MTase identification. For example, in *S*. Heidelberg CFSAN002064, we detected the gene for the putative methyltransferase Sen2064ORF15615P, and predicted that it would be responsible for GATC^m6^AG methylation. However, we did not observe the activity of this methyltransferase in *S*. Heidelberg, which means the enzyme is inactive. Inactivity can be the result of a mutation in the enzyme which renders it inactive, or, the enzyme may be functional, but not at the time of analysis. For example, some MTases may be inactive due to transcriptional silencing as is often found when the genes are present as part of a prophage [30]. Furthermore, an MTase may be transcribed, but for unknown reasons, may not routinely modify its’ target motif [12]. Cloning MTase genes has shown to be a useful approach for their characterization [6], and may help to match motifs to predicted MTases in cases where bioinformatics alone was insufficient. This approach should be incorporated into future studies that target particular MTases. For example, the cloning of Sen2064ORF15615P in an expression vector would resolve whether the enzyme is inactive or not functional in *S*. Heidelberg at the time of analysis.

We cannot completely rule out the possibility that DNA MTase genes exist that show no similarity to characterized MTase genes. However, with methylation data from more than 700 genomes available and almost 2,500 characterized and 50,000 putative MTase genes identified in REBASE, the chances of finding a completely new way of methylating DNA are getting increasingly smaller. In particular, we rarely come across a case where we can be certain that there are insufficient MTases to account for the observed patterns of methylation. However, in Salmonella enterica subsp. enterica serovar Heidelberg CFSAN002064, the methylated motif ACC^m6^ANCC occurs, which may indicate a plasmid is missing. This contrasts with CFSAN002069, which also has this motif, but does have a potential plasmid-encoded MTase. In other cases we have observed this motif is present in strains containing plasmids (R.J. Roberts, unpublished). Furthermore, as more genome sequence data and PacBio methylation data appear, our ability to predict recognition sequences from sequence data alone is growing. Already, rules are becoming apparent for predicting the specificity of Type IIG enzymes [31].

Most of the novel motifs observed in each serovar were modified by Type I RM systems (Fig 1). Type I systems have a modular structure that may allow sequence specificities to diversify more easily than the structures of other RM types (for review, see [32]). Each system consists of two methylase (M) units, two restriction endonuclease (R) units, and one sequence specificity (S) subunit [33, 34]. The S subunit has two TRDs, each of which recognizes one half of the target motif. Recombination events may occur on the S subunit, either within a single TRD or within the sequence that joins the two, resulting in novel specificity. Also, R and M subunits may interact with foreign S subunits entering the cell, also resulting in novel specificity. This has been observed in *Lactococcus* [35]. One interesting Type I motif, G^m6^ATGN_5_G^4m^ GC, is exhibited by the specificity subunit of the SenAboIII system. This example of cooperation between an m6A methylase and an m4C methylase is quite rare and has only been infrequently observed previously (R. Morgan, unpublished observations).

Unique motifs found among closely related taxa may be the result of horizontal gene transfer (HGT). Studies have demonstrated that HGT accounts for the movements of RM systems based on evidence of codon usage bias [36] and differential GC content of RM genes [37]. We identified several MTases that are located on prophages and plasmids, indicating possible mechanisms of transfer (Table 1). Also, through BLAST similarity searches against REBASE we found that several MTase sequences are most similar, or highly similar, to enzymes in Enterobacteriaceae genera other than *Salmonella*, suggesting that these systems may have been acquired via HGT. For example, M.SbaUII from *S*. Bareilly, which methylates the motif C^m6^AGCTG, is most similar to an MTase found in *Pectobacterium*. Currently, we are building a robust *Salmonella* phylogeny, including representatives of other Enterobacteriaceae genera, to test these and other evolutionary hypotheses.

In some taxa, we detected a proportion of motifs that were not fully methylated within the genome. In particular, only 38–78.5% of ATGC^m6^AT sites across the genome were methylated, and 89.3–100% of CAG^m6^AG sites were methylated (Table 2). Orphan MTases or RM systems with an inactive REase often do not methylate all sites in the genome, as complete methylation at all sites to protect from cleavage is usually unnecessary. Incomplete methylation may also be due to the fact that cells are analyzed at different times during the cell cycle, or methylation at certain sites may be inhibited by DNA binding proteins [38]. Environmental factors, including culture conditions, may also affect the frequency of methylation [9, 39]. Incomplete methylation may play a role in the regulation of gene expression. Thus, studies examining the functional implications of ATGC^m6^AT and CAG^m6^AG methylation will be particularly interesting.

In several of the genomes, ATGC^m6^AT methyltransferases are biased towards preferentially methylating this motif when preceded by a cytosine, a thymine, or both. For example, in *S*. Heidelberg CFSAN002069, AATGC^m6^AT and GATGC^m6^AT are methylated at lower frequencies than TATGC^m6^AT and CATGC^m6^AT. All four motifs are found in a roughly 1:1:1:1 ratio throughout the genome, indicating a true bias in methyltransferase activity. Currently, we are investigating the biological significance of these observations. Interestingly, 20 ATGC^m6^AT motifs are present in a collection of 101 previously characterized *Salmonella* virulence genes [40], and ten of these are AATGC^m6^ATs, a much higher proportion than what is expected by chance.

## Conclusions

In total, we observed 18 motifs among the nine *Salmonella* serovars, eight of which are novel. These findings indicate the diversity of motifs present in *Salmonella enterica*. The functions of the observed motifs are unknown, except for G^m6^ATC, which has been well studied and is involved in a variety of biological processes including virulence [15]. In *E*. *coli*, methylation of CTGC^m6^AG by the MTase M.EcoGIII, is shown to affect the transcription of over 30% of genes [12]. It is possible that the methylation of motifs in *Salmonella* described here may also play a role in virulence and other cell functions, and thus merit further study. Future studies should also continue to explore how methylation patterns vary across serovars, and examine within-serovar variation. Methylation may be useful as a typing marker, as closely related taxa are often difficult to differentiate using morphological and molecular markers. The reconstruction of a *Salmonella* phylogeny, along with the analysis of the methylomes will allow us to address these issues and gain a more broad view of the evolutionary history and functional significance of methylation within the genus.

## Supporting Information

S1 Fig Kinetic score (QV) vs. sequencing coverage for adenine residues in *S*. Heidelberg CFSAN002064. The line indicates the QV cutoff used for MTase specificity determination.
(TIF)

S1 Table Explanation of MTase assignments. (XLSX)

S2 Table SRA Accession numbers. (XLSX)
