# Supplementary figures and images for: Genome-Wide Methylation Patterns in Salmonella enterica Subsp. enterica Serovars
Source: PLoS One. 2015 Apr 10;10(4):e0123639. doi: 10.1371/journal.pone.0123639 (PMC4393132; doi:10.1371/journal.pone.0123639)

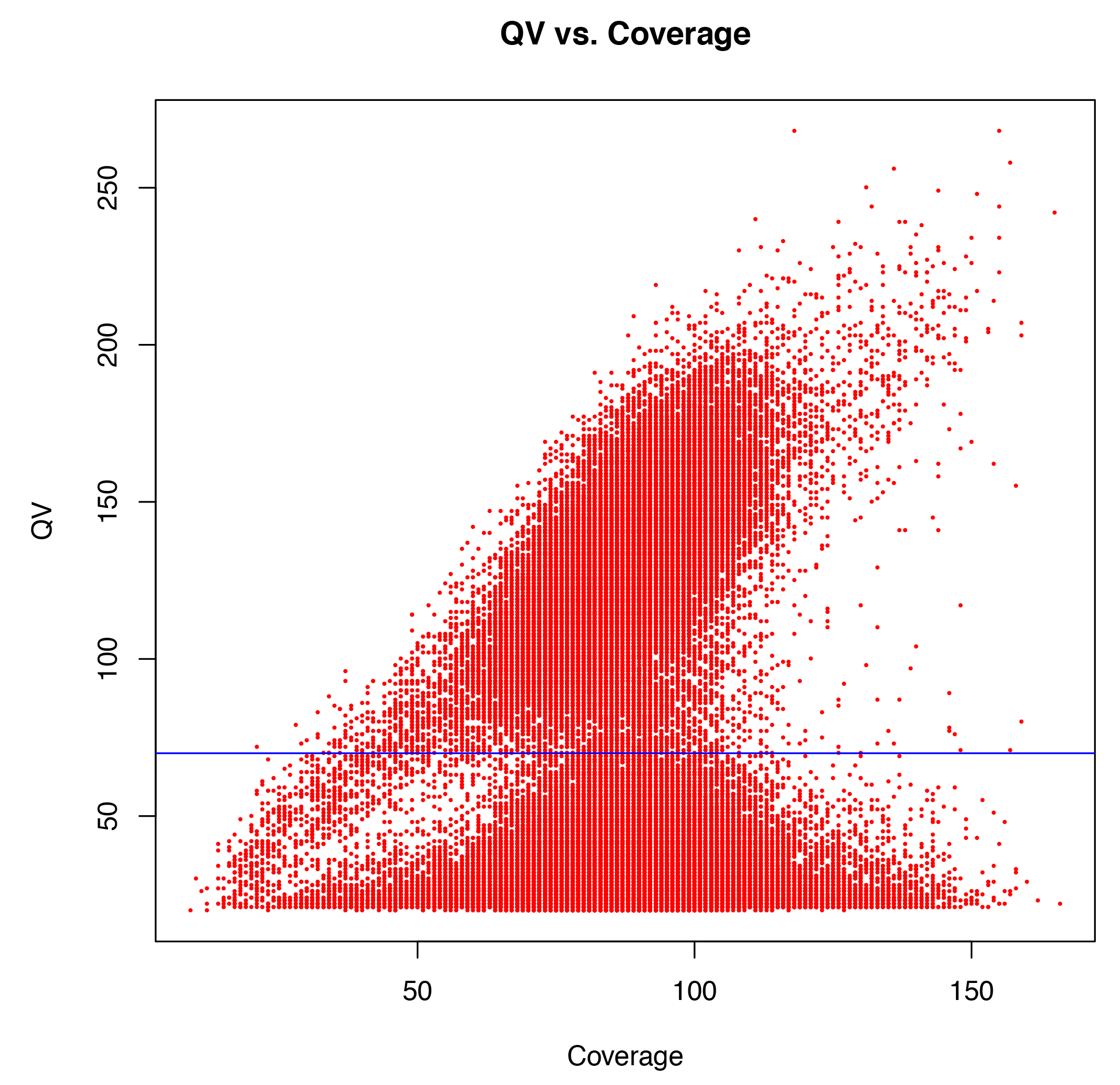

Supplement: S1 Fig — The line indicates the QV cutoff used for MTase specificity determination. (TIF) [file pone.0123639.s001.tif]
